# Differential activity of candidate microbicides against early steps of HIV-1 infection upon complement virus opsonization

**DOI:** 10.1186/1742-6405-7-16

**Published:** 2010-06-14

**Authors:** Mohammad-Ali Jenabian, Héla Saïdi, Charlotte Charpentier, Hicham Bouhlal, Dominique Schols, Jan Balzarini, Thomas W Bell, Guido Vanham, Laurent Bélec

**Affiliations:** 1 Université Paris Descartes (Paris V), Laboratoire de Virologie, Hôpital Européen Georges Pompidou, Paris, France; 2 Inserm U925, Laboratoire d'Immunologie, faculté de Médecine, Université Jules Verne Picardie, Amiens, France; 3 Rega Institute for Medical Research, Leuven, Belgium; 4 University of Nevada, Reno, NV, USA; 5 Virology Unit, Department of Microbiology, Institute of Tropical Medicine, Antwerpen, Belgium

## Abstract

**Background:**

HIV-1 in genital secretions may be opsonized by several molecules including complement components. Opsonized HIV-1 by complement enhances the infection of various mucosal target cells, such as dendritic cells (DC) and epithelial cells.

**Results:**

We herein evaluated the effect of HIV-1 complement opsonization on microbicide candidates' activity, by using three *in vitro *mucosal models: CCR5-tropic HIV-1_JR-CSF _transcytosis through epithelial cells, HIV-1_JR-CSF _attachment on immature monocyte-derived dendritic cells (iMDDC), and infectivity of iMDDC by CCR5-tropic HIV-1_BaL _and CXCR4-tropic HIV-1_NDK_. A panel of 10 microbicide candidates [T20, CADA, lectines HHA & GNA, PVAS, human lactoferrin, and monoclonal antibodies IgG1B12, 12G5, 2G12 and 2F5], were investigated using cell-free unopsonized or opsonized HIV-1 by complements. Only HHA and PVAS were able to inhibit HIV trancytosis. Upon opsonization, transcytosis was affected only by HHA, HIV-1 adsorption on iMDDC by four molecules (lactoferrin, IgG1B12, IgG2G5, IgG2G12), and replication in iMDDC of HIV-1_BaL _by five molecules (lactoferrin, CADA, T20, IgG1B12, IgG2F5) and of HIV-1_NDK _by two molecules (lactoferrin, IgG12G5).

**Conclusion:**

These observations demonstrate that HIV-1 opsonization by complements may modulate *in vitro *the efficiency of candidate microbicides to inhibit HIV-1 infection of mucosal target cells, as well as its crossing through mucosa.

## Background

Recent disappointing failure in microbicide clinical trials revealed that major gaps in basic and applied knowledges remain to conceive effective microbicide formulations [1-3]. In particular, the failure of phase II/III essays on candidate molecules having crossed successfully all the previous stages of the preclinical development, emphasizes the absolute necessity to establish a correlation between the preclinical criteria and the clinical criteria of microbicide molecules development [3]. Thus, one of the major objectives of *in vitro *evaluation of microbicide candidate molecules during their preclinical development is to get closer as much as possible to physiological conditions.

The inhibitory power of microbicide molecules may be affected by semen factors when male and female genital secretions are mixed during sexual intercourse, including pH, mucosal antibodies [4] and humoral soluble factors [5,6] For example, it has been recently demonstrated that the *in vitro *efficacy of polymeric microbicide molecules, acting as HIV-1 entry inhibitors, might become at least partly compromised by the presence of seminal plasma [7].

The system of the complement constitutes one of the first lines of innate defence. Its interaction with a multitude of pathogenic agents like viruses, leads its activation in cascade which ends in the deposit of C3 fragments on their surface. Unlike other pathogenic agents, the majority of HIV-1 particles escape the lysis by complement [8]. Free HIV-1 particles present in genital secretions may be likely opsonized by semen complement components [9-11]. Indeed, complement components are present in seminal fluid [9,11], and HIV by it-self is known to strongly activate the complement system [10]. We previously showed that opsonization of HIV-1 with complement enhanced infection of epithelial cells [12], and also enhanced infection of dendritic cells and viral transfer to CD4 T cells in a CR3 and DC-SIGN-dependent manner [13]. Thus, these findings support the hypothesis that the activity of microbicide molecules against HIV-1 may be influenced by the opsonization of the virus.

The aim of the present *proof-of-concept *study was to evaluate whether complement opsonization may affect the *in vitro *activity of a panel of microbicide molecule candidates acting against early steps of HIV-1 infection.

## Materials and methods

### Virus strains

Primary CCR5-tropic HIV-1_JR-CSF _and CXCR4-tropic HIV-1_NDK _were a gift from F. Barré-Sinoussi (Institut Pasteur, Paris). CCR5-tropic HIV-1_BaL _was provided by the National Institutes of Health (NIH, Maryland, USA). The viral stocks were amplified in monocyte-derived macrophages (MDM) of healthy donors and quantified by p24 capture ELISA measurements (DuPont de Nemours, France).

### Cells

Peripheral blood mononuclear cells (PBMC) were isolated from buffy coats of healthy adult donors by Ficoll density gradient centrifugation on Medium for Separation of Lymphocytes (MSL, Eurobio, Les Ulis, France), as previously described [14]. The percentage of monocytes was determined by flow cytometry using forward scatter and side scatter properties (FSC/SSC). PBMC were re-suspended in RPMI-1640 medium supplemented with L-glutamine, penicillin (100 IU/ml) and streptomycin (100 μg/ml). Cells were seeded into 24 well-plates (Costar, Cambridge, MA) at 10^6 ^adherent cells/ml, and incubated at 37°C for 45 min. Non-adherent cells were removed by 4 washes. Adherent monocytes were incubated in RPMI-1640 medium with 10% fetal calf serum (FCS), L-glutamine, and antibiotics. The relative concentration of rhM-CSF improved cell viability and maintained a neutral environment with respect to activation marker quantitative expression (HLA-DR, CD14, CD16), which remained similar to that of MDM cultured in medium alone. Immature monocyte-derived dendritic cells (iMDDC) were generated from monocytes in the presence of rhGM-CSF (10 ng/ml) in combination with rh-IL-4 (10 ng/ml). The medium, including all supplements, was replaced the third day of differentiation. After 6 days of culture, adherent cells corresponding to the dendritic cell-enriched fraction were harvested, washed, and used for subsequent experiments. Flow cytometry analysis (Becton Dickinson, NJ, USA) demonstrated that the dendritic cells were more than 90% pure.

The epithelial endometrial cell line HEC-1A was from the American Type Culture Collection [15], and was maintained in RPMI-1640 containing 10% FCS and antibiotics (100 μg of streptomycin per ml, and 100 IU of penicillin per ml).

### Candidate molecules

The gp120-interacting plant lectins *Hippeastrum hybrid *(amaryllis) (HHA) and *Galanthus nivalis *(snowdrop) (GNA) were derived and purified from the bulbs of these plants, as previously described [16]. The gp120-interacting sulfated polyvinyl alcohol (PVAS) which inhibits the virus entry, (molecular weight, 20,000 Da) was synthesized in the form of its sodium salts by the sulfation of PVA (polyvinyl alcohol) with chlorosulfonic acid in pyridine-dimethylformamide solution [17]. Human lactoferrin (Lf) [18] which limits the HIV-1 attachment on dendritic cells by inhibiting virus attachment on heparan sulfate proteoglycans [14] and mannan were obtained from Sigma-Aldrich (Saint-Louis, MO). CADA (cyclotriazadisulfonamide) [19] which inhibits the HIV entry by CD4 receptor down-modulating, was supplied by T.W. Bell (University of Nevada, Reno, NV) via the European Microbicides Project (EMPRO). The HIV-1 fusion inhibitor enfuvirtide (T20) and the HIV-1-specific neutralization monoclonal antibodies, IgG 2F5 directed to HIV-1 gp41, IgG 2G12 directed to HIV-1 carbohydrate side-chains of gp120, IgG 1B12 directed to the CD4 binding site of HIV-1 gp120 as well as IgG 12G5 directed to HIV-1 CXCR4 co-receptor, were obtained through the AIDS Reagent Program, Division of AIDS, NIAID, NIH.

Different concentrations of molecules dissolved in RPMI-1640 were used: Lectines HHA and GNA (1, 10 and 100 μg/ml), Lf (200 μg/ml), PVAS (1, 10 and 100 μg/ml), CADA (0.2 and 2 μg/ml), T20 (0.5 and 5 μg/ml), IgG2G12 (1 and 10 μg/ml), IgG2F5 (7.5 and 25 μg/ml), IgG12G5 (2.5 and 12.5 μg/ml), and IgG1B12 (1 and 10 μg/ml) and polyclonal anti-gp160 antibodies (10 μg/ml) as positive control for HIV inhibition.

### Complement opsonization of HIV-1

The activation of complement by HIV-1 and the generation of C3a Ag were similar by using a vol/vol ratio of human serum or seminal fluid, as previously demonstrated [12]. Thus, in our experimentations, human serum obtained from HIV-1-seronegative individuals was used as source of complement. Activation of serum complement and opsonization of free virus particles were carried out, as previously described [20]. Briefly, free HIV-1 (1 to 5 ng of HIV-1 p24 antigen) were added in a vol/vol ratio to serum supplemented with 0.6 mM CaCl_2 _and 0.9 mM MgCl_2 _for 1 h at 37°C in order to initialize the complement activation by viral particles (Ops). As negative controls, serum was heat-inactivated by incubation for 1 h at 56°C, and added to viral particles in similar conditions to obtain heat-inactivated non-opsonized free HIV-1 (HI NonOps). Negative control corresponding to non-opsonized HIV-1 (NonOps) was obtained by HIV-1 incubation in culture medium for 1 h at 37°C.

### Inhibition of HIV-1 transcytosis

HEC-1A cells were grown on a 0.4 μm-pore polycarbonate permeable support (Transwell, Costar, MA), as previously described [21]. Tightness of the monolayer of HEC-1A cells was monitored by measuring resistance at day 6 of culture that must have reached 300 Ω/cm^2^. Increasing concentrations of microbicide molecules and HIV-1 (5 ng of HIV-1 p24 antigen) pre-incubated with complement or heat-inactivated complement were then added to the apical side of HEC-1A for 3h at 37°C. HIV-1 transcytosis was assessed by measuring the p24 antigen concentration in the basolateral chamber medium by p24 antigen capture ELISA. Positive control for transcytosis consisted of free HIV-1. Positive control for transcytosis inhibition consisted of free HIV-1 incubated 30 min with purified polyclonal antibodies directed to HIV-1 gp160 before to be added to the apical side of the HEC-1A cell cultures, as described [21].

### Inhibition of HIV-1 adsorption on dendritic cells

Complement-Ops or NonOps HIV-1 (1 ng of HIV-1 p24 antigen) were incubated with iMDDC (10^5 ^cells/well) in the presence of increasing concentrations of microbicide molecules for 1 h at 37°C. After 4 washes to remove unattached virus, cells were lysed by adding PBS 1% Triton X-100 for 45 min at 37°C, and the concentration of HIV-1 p24 antigen was measured [14]. Polyclonal purified antibodies to gp160 and mannan were used as positive controls.

### Inhibition of iMDDC infection by HIV-1

Cells were washed 2 times after 6 days of differentiation and seeded into 96-well culture plates (5 × 10^5 ^cells per well). Complement-Ops or NonOps HIV-1 (1 ng p24 antigen/ml) and increasing concentrations of microbicide candidate molecules were added on cells and incubated for 3 h at 37°C in a 5% CO_2 _atmosphere. Each sample was performed in triplicate. After 4 washes to remove exceeding virus, cells were cultured for 3 days. The amounts of virus replication were monitored by HIV-1 p24 antigen ELISA. In this last case, supernatants were harvested and virus particles were lysed by incubation for 45 min at 37°C with 1% Triton X-100. Polyclonal purified antibodies to gp160 were used as positive controls.

### Statistical analysis

Mann-Whitney U-test was used for statistical analysis, with P < 0.05 being considered as significant.

## Results

### HIV-1 transcytosis inhibition by microbicide candidate molecules upon HIV-1 complement opsonization

HIV-1_JR-CSF _was incubated with increasing compound concentrations before to be added to the apical membrane of HEC-1A cells. As shown in Table 1, the transcytosis of NonOps HIV-1 and that of HI NonOps HIV-1 were inhibited in a dose-dependent manner by HHA. In contrast, HHA had no effect on transcytosis of Ops HIV-1. Thus, HHA lost its ability to block HIV-1 transcytosis when the virus was opsonized by complement components. PVAS inhibited transcytosis of Ops HIV-1, HI NonOps HIV-1 and NonOps HIV-1. For a given concentration, PVAS inhibited with the same efficiency the transcytosis of Ops HIV-1 and that of NonOps HIV-1. The other microbicide candidates did not interfere with the transcytosis of Ops HIV-1, HI NonOps HIV-1, and NonOps HIV-1. As positive control for HIV-1 transcytosis inhibition, polyclonal anti-gp160 antibodies (10 μg/ml) inhibited at 90% the transcytosis of Ops HIV-1, HI NonOps HIV-1 and NonOps HIV-1. Free Ops HIV-1, HI NonOps HIV-1 and NonOps HIV-1, not incubated with microbicide molecules nor with anti-gp160 antibodies, were capable to be transcytosed through HEC-1 cells with identical rates. Similar results were obtained when Ops HIV-1, HI NonOps HIV-1 and NonOps HIV-1 were incubated with irrelevant immunoglobulins (not shown). In summary, upon complement opsonization, transcytosis blocking was abolished for 1 molecule (HHA).

**Table 1:** Inhibition by microbicide molecule candidates of the transcytosis of HIV-1_JR-CSF _through a tight monolayer of endometrial epithelial HEC-1A cells

	NonOps	HI NonOps	Ops
**HHA**	I (54%)*	I (48%)	NI [S]**

**GNA**	NI	NI	NI

**PVAS**	I (65%)	I (63%)	I (65%)

**Lf**	NI	NI	NI

**CADA**	NI	NI	NI

**T20**	NI	NI	NI

**IgG1B12**	NI	NI	NI

**IgG12G5**	NI	NI	NI

**IgG2G12**	NI	NI	NI

**IgG2F5**	NI	NI	NI

Ab to gp160***	I (90%)	I (90%)	I (90%)

* Percentage of transcytosis inhibition in brackets

** Significant difference between the percentages of transcytosis inhibition according to Ops, HI NonOps and NonOps HIV-1 (Mann & Whitney *U *test)

*** Used as positive control

NonOps: Non opsonized free HIV-1; HI NonOps: Heat inactivated non opsonized free HIV-1; Ops: Free HIV-1 opsonized virus by complement components

I: Transcytosis inhibition; NI: Lack of transcytosis inhibition

S: Significant

The transcytosis inhibition is shown for the best doses of the candidate molecules, and is expressed as percentage of the average of three independent experiments. The range of detected HIV-1 p24 antigen for uninhibited transcytosis in negative control experimentation (without microbicide molecules) was 150-210 pg/ml.

### Inhibition of HIV-1 adsorption on iMDDC by the microbicide molecules upon HIV-1 complement opsonization

HHA and GNA lectins at concentrations of 100 μg/ml inhibited the adsorption of Ops HIV-1, NonOps HIV-1 and HI NonOps HIV-1 on iMDDC with similar efficiencies (Table 2). Lf and the monoclonal antibodies IgG1B12, IgG12G5, and IgG2F5 showed differential effect on the inhibition of HIV-1 adsorption on iMDDC according to complement opsonization of the virus. Thus, they had no effect on Ops HIV-1 attachment to iMDDC, whereas they inhibited the attachment of NonOps HIV-1 as well as HI NonOps HIV-1. PVAS, CADA and T20 did not interfere with the attachment of Ops and NonOps HIV-1 on iMDDC. Both positive controls, polyclonal antibodies to gp160 (10 μg/ml) and mannan (250 μg/ml), inhibited the adsorption of NonOps and HI NonOps HIV-1, but their inhibiting capacities decreased for Ops HIV-1 (Table 2). In summary, upon complement opsonization, HIV-1 adsorption on iMDDC was counteracted by four molecules (Lf, IgG1B12, IgG2G5, IgG2G12).

**Table 2:** Inhibition of the adsorption of HIV-1_JR-CSF _on immature monocyte-derived dendritic cells by microbicide molecule candidates

	NonOps	HI NonOps	Ops
**HHA**	I (47%)*	I (43%)	I (48%)

**GNA**	I (47%)	I (43%)	I (48%)

**PVAS**	NI	NI	NI

**Lf**	I (37%)	I (32%)	NI [S]**

**CADA**	NI	NI	NI

**T20**	NI	NI	NI

**IgG1B12**	I (28%)	I (14%)	NI [S]

**IgG12G5**	I (26%)	I (16%)	NI [S]

**IgG2G12**	I (19%)	I (16%)	NI [S]

**IgG2F5**	I (17%)	I (17%)	I (11%)

Mannan***	I (42%)	I (33%)	NI [S]

Ab to gp160***	I (52%)	I (47%)	I (21%) [S]

* Percentage of inhibition of virus adsorption on dendritic cells in brackets

** Significant difference between the percentages of adsorption inhibition according to Ops, HI NonOps and NonOps HIV-1 (Mann & Whitney *U *test)

*** Used as positive controls

NonOps: Non opsonized free HIV-1; HI NonOps: Heat inactivated non opsonized free HIV-1; Ops: Free HIV-1 opsonized virus by complement components

I: Inhibition of virus adsorption on dendritic cells; NI: Lack of inhibition of virus adsorption on dendritic cells

S: Significant

The adsorption inhibition is shown for the optimal doses of the candidate molecules, and is expressed as percentage of the average of three independent experiments. The range of detected HIV-1 p24 antigen for uninhibited adsorption in negative control experimentation (without microbicide molecules) was 200-500 pg/ml. The capability of dendritic cells to capture HIV is donor-dependent.

### HIV-1 replication in iMDDC by the microbicide molecules upon HIV-1 complement opsonization

The molecules Lf, CADA, T20, IgG1B12, IgG2G12 and IgG2F5 inhibited the iMDDC infection by NonOps HIV-1_BaL _in a dose-dependent manner (Table 3). These latter molecules were able to inhibit the Ops HIV-1_BaL_, but their inhibiting capacity was decreased, except for IgG2G12 which inhibited both Ops and NonOps HIV-1 to a similar extent. IgG12G5 did not have any effect when the cells were infected by HIV-1_BaL_. The molecules Lf, CADA, T20 and all tested monoclonal antibodies, inhibited the replication of NonOps HIV-1_NDK _in iMDDC in a dose-dependent manner. Similarly, they were able to inhibit Ops HIV-1 without reduction in their inhibitory capacities, except Lf and IgG12G5. The polyclonal antibodies to gp160, used as positive control, inhibited the infection of dendritic cells by Ops, HI NonOps and NonOps HIV-1_BaL _or HIV-1_NDK_, at 50-61% and 69-91%, respectively (Table 3). In summary, upon complement opsonization, replication of HIV-1_BaL _in iMDDC was changed for 5 molecules (Lf, CADA, T20, IgG1B12, IgG2F5) and that of HIV-1_NDK _for 2 molecules (Lf, IgG12G5).

**Table 3:** Inhibition of the production of HIV-1_BaL _or HIV-1_NDK _in immature monocyte-derived dendritic cells by microbicide molecule candidates

	**HIV-1**_**BaL**_			**HIV-1**_**NDK**_		
	**NonOps**	**HI NonOps**	**Ops**	**NonOps**	**HI NonOps**	**Ops**

**Lf**	I (30%)*	I (26%)	NI [S]**	I (63%)	I (61%)	I (26%) [S]

**CADA**	I (61%)	I (64%)	NI [S]	I (95%)	I (93%)	I (96%)

**T20**	I (83%)	I (82%)	I (57%) [S]	I (95%)	I (95%)	I (97%)

**IgG1B12**	I (86%)	I (85%)	I (60%) [S]	I (94%)	I (96%)	I (94%)

**IgG12G5**	NI	NI	NI	I (95%)	I (95%)	I (54%) [S]

**IgG2G12**	I (67%)	I (62%)	I (69%)	I (96%)	I (95%)	I (96%)

**IgG2F5**	I (84%)	I (77%)	I (41%) [S]	I (97%)	I (96%)	I (97%)

Ab to gp160***	I (61%)	I (58%)	I (50%) [S]	I (79%)	I (91%)	I (69%)

* Percentage of virus production inhibition in brackets

** Significant difference between the percentages of production inhibition is significant according to Ops, HI NonOps and NonOps HIV-1 (Mann & Whitney *U *test)

*** Used as positive control

NonOps: Non opsonized free HIV-1; HI NonOps: Heat inactivated non opsonized free HIV-1; Ops: Free HIV-1 opsonized virus by complement components

I: Virus production inhibition; NI: Lack of transcytosis inhibition

S: Significant

The infection inhibition is shown for the best doses of the candidate molecules, and is expressed as percentage of the average of three independent experiments. The range of detected HIV-1 p24 antigen for uninhibited HIV-1 replication in negative control experimentations (without microbicide molecules) was 800-1000 pg/ml in iMDDC infectivity assay. The capability of dendritic cells to replicate HIV-1 is donor-dependent.

## Discussion

The present *proof-of-concept *study was conceived to evaluate the influence of HIV-1 opsonization by complement components on the inhibition of HIV-1 transcytosis through a monolayer of human endometrial epithelial cells, HIV-1 capture by dendritic cells, and HIV-1 productive infection of dendritic cells by a panel of 10 microbicide candidate molecules. Upon complement opsonization, transcytosis blocking was changed by 1 molecule (HHA), HIV-1 adsorption on iMDDC for 4 molecules (Lf, IgG1B12, IgG2G5, IgG2G12), and replication in iMDDC of HIV-1_BaL _by 5 molecules (Lf, CADA, T20, IgG1B12, IgG2F5) and of HIV-1_NDK _by 2 molecules (Lf, IgG12G5). These findings clearly demonstrate that HIV-1 opsonization by complement components may modulate *in vitro *the efficiency of microbicide candidate molecules to inhibit HIV-1 infection of potential mucosal target cells, as well as the crossing of the virus through mucosa. Since complement is present in male genital fluid, these observations allow to make the hypothesis that semen complement opsonization of HIV-1 could modulate *in vivo *the anti-HIV-1 activity of microbicides.

Among several factors possibly involved in the modulation of microbicide activity by seminal plasma, we focused on complement components. Indeed, complement components have been detected in all body secretions, including seminal fluid [9,11]. Since HIV is known to activate complement system [10], HIV-1 particles in male genital secretions may be likely opsonized by semen components. Activation of complement by HIV-1 results in deposition of C3 fragments on the viral surface without formation of complement lysis complex [22,23], resulting in opsonized HIV-1 harboring complement components covalently linked to the surface viral glycoproteins, and thus changing the virus phenotype [8,22,24-29]. In addition, opsonization of HIV-1 with complement modulates *in vitro *the infection of epithelial [12] and dendritic cells[13], as well as the transfer of HIV-1 from dendritic cells to CD4 T cells [13].

We first evaluated the ability of each molecule to inhibit HIV-1 transcytosis through a monolayer of epithelial cells [21,30] in the presence or absence of HIV-1 opsonization by complement. The HIV-1_JR-CSF _strain was exclusively used in our transcytosis assays, because transcytosis was shown to be selective, the HIV-1_BaL_. strain being not able to cross the monolayer of HEC1 epithelial cells [21]. Both free NonOps and Ops HIV-1 were similarly transcytosed. HHA and PVAS molecules limited efficiently NonOps HIV-1 transcytosis. Incubation of Ops HIV-1 with HHA or PVAS resulted in a complete loss of the ability of HHA to block transcytosis, whereas PVAS remained efficient. Indeed, the mannose-specific lectin HHA may inhibit HIV-1 entry into its target cells by interacting with the heavily glycosylated gp120 envelope glycoprotein [16,31,32]. In parallel, high-mannose-binding complement fragments interact with gp120 [4]. Thus, HHA-binding sites on gp120 may be hidden by complement molecules when the virus is opsonized. In contrast to HHA, GNA did not interfere with NonOps and Ops HIV-1 transcytosis. GNA has predominant specificity for α(1-3)-linked mannose residues whereas HHA can recognize both α(1-3)- and α(1-6)-linked mannose residues [27]. The differential effect observed for these two lectins in association with the lack of HIV-1 transcytosis inhibition by HHA when the virus is opsonized, suggests that free α(1-6)-linked mannose residues are no more accessible at the surface of complement opsonized virus. PVAS inhibited HIV-1 transcytosis independently of the virus opsonization. PVAS is a polyanionic molecule that may exert its activity against HIV-1 by shielding-off the positively charged aminoacid residues on the V3/gp120 loop [17], thus preventing the interaction of gp120 with heparan sulfated proteoglycans (HSPG) which are largely expressed on epithelial cells and involved in HIV-1 adsorption [33,34]. Complement opsonization of HIV-1 was not able to prevent PVAS inhibitory activity, suggesting that opsonization does not modify the positively charged HIV-1 surface glycoproteins and that the PVAS target site on gp120 could be reachable even in the presence of complement components.

We further investigated whether opsonization of HIV-1 may modulate the capability of microbicide molecules to inhibit HIV-1 adsorption on dendritic cells. HIV-1 opsonization enhanced by 50% viral adsorption on dendritic cells as compared with NonOps HIV-1, as previously reported [13]. Such increased binding of HIV-1 could be explained by the expression on dendritic cells of complement receptors (CR3). Increased binding of HIV-1 could facilitate the infection of dendritic cells since complement is considered as an enhancer of HIV-1 infection [12,13,20,22,23,25]. HHA and GNA inhibited with the same efficiency NonOps and Ops HIV-1 adsorption. PVAS, which hampers the interaction between HIV-1 and HSPG expressed on target cells, had no effect on HIV-1 adsorption on dendritic cells, likely because these cells express only slightly HSPG. Since plant lectins do not interfere with DC-SIGN [36], a mannose receptor largely expressed on dendritic cells [32], our observations suggest that other mannose receptors than DC-SIGN may be involved in HIV-1 adsorption on dendritic cells, as previously reported [37,38]. One hypothesis could be that NonOps and Ops HIV-1 interact principally with surface proteins exhibiting terminal α(1-3)-mannosylation on dendritic cells. In contrast, PVAS had no effect on HIV-1 adsorption on dendritic cells. Mannan, a major mannose binding proteins ligand, inhibited adsorption of NonOps virus, but was less effective by using the Ops HIV-1. This phenomenon confirms then that NonOps and Ops viruses may use different receptors involved in their adsorption at the surface of dendritic cells, as previously reported [13].

The HIV-1-specific monoclonal antibodies were able to inhibit the NonOps HIV-1, but not Ops HIV-1. The virus opsonized by complement fragments uses the complement receptor type 3 (CR3) for its adsorption on dendritic cells [13]. Thus, the viral glycoproteins gp41 and gp120 are likely less used for virus adsorption on dendritic cells in presence of complement compounds, as strongly suggested by the less efficiency of polyclonal antibodies to gp160 to inhibit the adsorption of Ops HIV-1 at the surface of dendritic cells. Lf was no more able to inhibit HIV-1 adsorption on dendritic cells when the virus was opsonized. This latter finding suggests the existence of a Lf-binding site hidden by complement components. Lf may prevent NonOps HIV-1 adsorption on dendritic cells by cell receptors not used by Ops HIV-1, like nucleolin involved in the adsorption on the cellular membrane of both Lf and HIV-1 [39,40]. Taken together, the possibility exists that semen complement opsonization of HIV-1 in human genital secretions may allow the virus to escape to the antiviral activity of natural inhibitors such as Lf.

Finally, we evaluated the role of opsonization on dendritic cell infection. The monoclonal antibody IgG12G5, an inhibitor of CXCR4 coreceptor, had no effect on the replication of CCR5-tropic HIV-1. All tested molecules inhibited the infection of dendritic cells by CCR5- and CXCR4- tropic NonOps HIV-1. By using the CCR5-tropic Ops HIV-1_BaL_, we observed that the inhibitory effects of the microbicide candidates significantly decreased in the presence of the complement. In contrast, the molecules inhibited the CXCR4-tropic Ops HIV-1_NDK _with the same efficiency as the NonOps HIV-1_NDK_, except for Lf and IgG 12G5. According to the hypothesis proposed by Margolis & Shattock [41], the CCR5-tropic viral strains may be selected during the sexual transmission of HIV-1 and in the early stages of infection by HIV-1. Our observations indicate that some microbicide molecules may be less inhibitory against CCR5-tropic HIV-1 when the virus is opsonized by complement components, and thus could be less efficient in early infection of dendritic cells.

In conclusion, virus complement opsonization may modulate the inhibitory activity of microbicide molecules against HIV *in vitro*, and could be also involved *in vivo *as possible modulatory factor of their anti-HIV-1-inhibitory activities when the drugs are mixed with male genital secretions containing high concentrations of complement. Microbicide candidate molecules whose *in vitro *anti-HIV activity is not influenced, or positively reinforced, by complement opsonisation of HIV, could be likely retained for further steps of preclinical development. However, the hypothesis that seminal complement components could *in vivo *modulate the inhibitory activities of several microbicide candidate molecules acting at different targets against the virus, warrants further investigations.

## Competing interests

The authors declare that they have no competing interests.

## Authors' contributions

MAJ, HS, CC and HB performed the experiments. MAJ, HS and LB analyzed data and wrote the paper. JB and DS participated in the design of the study and provided HHA and GNA. TWB provided CADA and helped draft the manuscript. GV participated in the design and coordination of the study and helped draft the manuscript. LB conceived the study, participated in its design and coordination, analyzed data and wrote the paper.

All authors read and approved the final manuscript.

## References

[B1] Feldblum PJ Adeiga A Bakare R Wevill S Lendvay A Obadaki F Olayemi MO Wang L Nanda K Rountree W SAVVY vaginal gel (C31G) for prevention of HIV infection: a randomized controlled trial in Nigeria PloS one 2008 3 1 e1474 10.1371/journal.pone.0001474 18213382 PMC2190795

[B2] Van Damme L Govinden R Mirembe FM Guedou F Solomon S Becker ML Pradeep BS Krishnan AK Alary M Pande B Lack of effectiveness of cellulose sulfate gel for the prevention of vaginal HIV transmission The New England journal of medicine 2008 359 5 463 472 10.1056/NEJMoa0707957 18669425

[B3] Saidi H Jenabian MA Bélec L Early events in vaginal HIV transmission: Implications in microbicide development Future Virol 2009 4 3 259 269 10.2217/fvl.09.10

[B4] Ying H Ji X Hart ML Gupta K Saifuddin M Zariffard MR Spear GT Interaction of mannose-binding lectin with HIV type 1 is sufficient for virus opsonization but not neutralization AIDS research and human retroviruses 2004 20 3 327 335 10.1089/088922204322996563 15117456

[B5] Royce RA Sena A Cates W Jr Cohen MS Sexual transmission of HIV The New England journal of medicine 1997 336 15 1072 1078 10.1056/NEJM199704103361507 9091805

[B6] Shepard RN Schock J Robertson K Shugars DC Dyer J Vernazza P Hall C Cohen MS Fiscus SA Quantitation of human immunodeficiency virus type 1 RNA in different biological compartments Journal of clinical microbiology 2000 38 4 1414 1418 10747117 10.1128/jcm.38.4.1414-1418.2000 PMC86455

[B7] Neurath AR Strick N Li YY Role of seminal plasma in the anti-HIV-1 activity of candidate microbicides BMC infectious diseases 2006 6 150 10.1186/1471-2334-6-150 17042959 PMC1618840

[B8] Stoiber H Speth C Dierich MP Role of complement in the control of HIV dynamics and pathogenesis Vaccine 2003 21 Suppl 2 S77 82 10.1016/S0264-410X(03)00203-2 12763687

[B9] Rahimi A Sepehri H Pakravesh J Bahar K Quantification of C3 and C4 in infertile men with antisperm antibody in their seminal plasma Am J Reprod Immunol 1999 41 5 330 336 10378028 10.1111/j.1600-0897.1999.tb00446.x

[B10] Stoiber H Banki Z Wilflingseder D Dierich MP Complement-HIV interactions during all steps of viral pathogenesis Vaccine 2008 26 24 3046 3054 10.1016/j.vaccine.2007.12.003 18191309

[B11] Vanderpuye OA Labarrere CA McIntyre JA The complement system in human reproduction Am J Reprod Immunol 1992 27 3-4 145 155 1384536 10.1111/j.1600-0897.1992.tb00742.x

[B12] Bouhlal H Chomont N Haeffner-Cavaillon N Kazatchkine MD Belec L Hocini H Opsonization of HIV-1 by semen complement enhances infection of human epithelial cells J Immunol 2002 169 6 3301 3306 12218150 10.4049/jimmunol.169.6.3301

[B13] Bouhlal H Chomont N Requena M Nasreddine N Saidi H Legoff J Kazatchkine MD Belec L Hocini H Opsonization of HIV with complement enhances infection of dendritic cells and viral transfer to CD4 T cells in a CR3 and DC-SIGN-dependent manner J Immunol 2007 178 2 1086 1095 17202372 10.4049/jimmunol.178.2.1086

[B14] Saidi H Eslahpazir J Carbonneil C Carthagena L Requena M Nassreddine N Belec L Differential modulation of human lactoferrin activity against both R5 and X4-HIV-1 adsorption on epithelial cells and dendritic cells by natural antibodies J Immunol 2006 177 8 5540 5549 17015741 10.4049/jimmunol.177.8.5540

[B15] Ball JM Moldoveanu Z Melsen LR Kozlowski PA Jackson S Mulligan MJ Mestecky JF Compans RW A polarized human endometrial cell line that binds and transports polymeric IgA In vitro cellular & developmental biology 1995 31 3 196 206 10.1007/BF02639434 7757302

[B16] Van Damme EJAA Peumans WJ Related mannose-specific lectins from different species of the family Amaryllidaceae Physiol Plant 1988 73 52 57 10.1111/j.1399-3054.1988.tb09192.x

[B17] Baba M Schols D De Clercq E Pauwels R Nagy M Gyorgyi-Edelenyi J Low M Gorog S Novel sulfated polymers as highly potent and selective inhibitors of human immunodeficiency virus replication and giant cell formation Antimicrobial agents and chemotherapy 1990 34 1 134 138 2327749 10.1128/aac.34.1.134 PMC171534

[B18] Berkhout B Floris R Recio I Visser S The antiviral activity of the milk protein lactoferrin against the human immunodeficiency virus type 1 Biometals 2004 17 3 291 294 10.1023/B:BIOM.0000027707.82911.be 15222480

[B19] Vermeire K Brouwers J Van Herrewege Y Le Grand R Vanham G Augustijns P Bell TW Schols D CADA, a potential anti-HIV microbicide that specifically targets the cellular CD4 receptor Current HIV research 2008 6 3 246 256 10.2174/157016208784324958 18473788

[B20] Bouhlal H Galon J Kazatchkine MD Fridman WH Sautes-Fridman C Haeffner Cavaillon N Soluble CD16 inhibits CR3 (CD11b/CD18)-mediated infection of monocytes/macrophages by opsonized primary R5 HIV-1 J Immunol 2001 166 5 3377 3383 11207294 10.4049/jimmunol.166.5.3377

[B21] Hocini H Becquart P Bouhlal H Chomont N Ancuta P Kazatchkine MD Belec L Active and selective transcytosis of cell-free human immunodeficiency virus through a tight polarized monolayer of human endometrial cells Journal of virology 2001 75 11 5370 5374 10.1128/JVI.75.11.5370-5374.2001 11333919 PMC114943

[B22] Stoiber H Frank I Spruth M Schwendinger M Mullauer B Windisch JM Schneider R Katinger H Ando I Dierich MP Inhibition of HIV-1 infection in vitro by monoclonal antibodies to the complement receptor type 3 (CR3): an accessory role for CR3 during virus entry? Molecular immunology 1997 34 12-13 855 863 10.1016/S0161-5890(97)00108-9 9464521

[B23] Thieblemont N Delibrias C Fischer E Weiss L Kazatchkine MD Haeffner-Cavaillon N Complement enhancement of HIV infection is mediated by complement receptors Immunopharmacology 1993 25 2 87 93 10.1016/0162-3109(93)90012-F 8500986

[B24] Banki Z Stoiber H Dierich MP HIV and human complement: inefficient virolysis and effective adherence Immunology letters 2005 97 2 209 214 10.1016/j.imlet.2004.11.007 15752560

[B25] Pinter C Siccardi AG Lopalco L Longhi R Clivio A HIV glycoprotein 41 and complement factor H interact with each other and share functional as well as antigenic homology AIDS research and human retroviruses 1995 11 8 971 980 10.1089/aid.1995.11.971 7492444

[B26] Schmitz J Zimmer JP Kluxen B Aries S Bogel M Gigli I Schmitz H Antibody-dependent complement-mediated cytotoxicity in sera from patients with HIV-1 infection is controlled by CD55 and CD59 The Journal of clinical investigation 1995 96 3 1520 1526 10.1172/JCI118190 7544808 PMC185777

[B27] Stoiber H Ammann C Spruth M Mullauer B Eberhart A Harris CL Huber CG Longhi R Falkensammer B Wurzner R Enhancement of complement-mediated lysis by a peptide derived from SCR 13 of complement factor H Immunobiology 2001 203 4 670 686 11402501 10.1016/s0171-2985(01)80016-4

[B28] Stoiber H Pinter C Siccardi AG Clivio A Dierich MP Efficient destruction of human immunodeficiency virus in human serum by inhibiting the protective action of complement factor H and decay accelerating factor (DAF, CD55) The Journal of experimental medicine 1996 183 1 307 310 10.1084/jem.183.1.307 8551237 PMC2192395

[B29] Takefman DM Sullivan BL Sha BE Spear GT >Mechanisms of resistance of HIV-1 primary isolates to complement-mediated lysis Virology 1998 246 2 370 378 10.1006/viro.1998.9205 9657955

[B30] Stone A Microbicides: a new approach to preventing HIV and other sexually transmitted infections Nature reviews 2002 1 12 977 985 10.1038/nrd959 12461519

[B31] Balzarini J Targeting the glycans of glycoproteins: a novel paradigm for antiviral therapy Nat Rev Microbiol 2007 5 8 583 597 10.1038/nrmicro1707 17632570 PMC7098186

[B32] Balzarini J Hatse S Vermeire K Princen K Aquaro S Perno CF De Clercq E Egberink H Vanden Mooter G Peumans W Mannose-specific plant lectins from the Amaryllidaceae family qualify as efficient microbicides for prevention of human immunodeficiency virus infection Antimicrobial agents and chemotherapy 2004 48 10 3858 3870 10.1128/AAC.48.10.3858-3870.2004 15388446 PMC521907

[B33] Saidi H Magri G Nasreddine N Requena M Belec L R5- and X4-HIV-1 use differentially the endometrial epithelial cells HEC-1A to ensure their own spread: implication for mechanisms of sexual transmission Virology 2007 358 1 55 68 10.1016/j.virol.2006.07.029 16934308

[B34] Vives RR Imberty A Sattentau QJ Lortat-Jacob H Heparan sulfate targets the HIV-1 envelope glycoprotein gp120 coreceptor binding site The Journal of biological chemistry 2005 280 22 21353 21357 10.1074/jbc.M500911200 15797855

[B35] June RA Schade SZ Bankowski MJ Kuhns M McNamara A Lint TF Landay AL Spear GT Complement and antibody mediate enhancement of HIV infection by increasing virus binding and provirus formation AIDS (London, England) 1991 5 3 269 274 1829367 10.1097/00002030-199103000-00004

[B36] Geijtenbeek TB Kwon DS Torensma R van Vliet SJ van Duijnhoven GC Middel J Cornelissen IL Nottet HS KewalRamani VN Littman DR DC-SIGN, a dendritic cell-specific HIV-1-binding protein that enhances trans-infection of T cells Cell 2000 100 5 587 597 10.1016/S0092-8674(00)80694-7 10721995

[B37] Requena M Bouhlal H Nasreddine N Saidi H Gody JC Aubry S Gresenguet G Kazatchkine MD Sekaly RP Belec L Inhibition of HIV-1 transmission in trans from dendritic cells to CD4+ T lymphocytes by natural antibodies to the CRD domain of DC-SIGN purified from breast milk and intravenous immunoglobulins Immunology 2008 123 4 508 518 10.1111/j.1365-2567.2007.02717.x 17999675 PMC2433318

[B38] Saidi H Magri G Carbonneil C Nasreddine N Requena M Belec L >IFN-gamma-activated monocytes weakly produce HIV-1 but induce the recruitment of HIV-sensitive T cells and enhance the viral production by these recruited T cells Journal of leukocyte biology 2007 81 3 642 653 10.1189/jlb.0406278 16971466

[B39] Legrand D Vigie K Said EA Elass E Masson M Slomianny MC Carpentier M Briand JP Mazurier J Hovanessian AG Surface nucleolin participates in both the binding and endocytosis of lactoferrin in target cells European journal of biochemistry/FEBS 2004 271 2 303 317 10.1046/j.1432-1033.2003.03929.x 14717698

[B40] Nisole S Said EA Mische C Prevost MC Krust B Bouvet P Bianco A Briand JP Hovanessian AG The anti-HIV pentameric pseudopeptide HB-19 binds the C-terminal end of nucleolin and prevents anchorage of virus particles in the plasma membrane of target cells The Journal of biological chemistry 2002 277 23 20877 20886 10.1074/jbc.M110024200 11919179

[B41] Margolis L Shattock R Selective transmission of CCR5-utilizing HIV-1: the 'gatekeeper' problem resolved? Nat Rev Microbiol 2006 4 4 312 317 10.1038/nrmicro1387 16541138

